# Copper(II)-β-cyclodextrin immobilized on graphitic carbon nitride nanosheets as a highly effective catalyst for tandem oxidative amidation of benzylic alcohols

**DOI:** 10.1038/s41598-022-05363-z

**Published:** 2022-02-11

**Authors:** Hossein Ghafuri, Afsaneh Rashidizadeh, Mostafa Ghafori Gorab, Ghazaleh Jafari

**Affiliations:** Catalysts and Organic Synthesis Research Laboratory, Department of Chemistry, Iran University of Science and Technology, 16846-13114 Tehran, Iran

**Keywords:** Catalyst synthesis, Heterogeneous catalysis

## Abstract

In this study, an efficient catalyst based on graphitic carbon nitride nanosheets (CN) and copper(II) supported β-cyclodextrin (β-CD/Cu(II)) was synthesized and used for tandem oxidative amidation of benzylic alcohols. In this regard, CN was functionalized by β-CD/Cu(II) via 1,3-dibromopropane linker (CN-Pr-β-CD/Cu(II)). The prepared catalyst was characterized using FT-IR, XRD, FE-SEM, EDS, TGA, ICP-OES, BET, and TEM analyses. CN-Pr-β-CD/Cu(II) was subsequently applied in a direct oxidative amidation reaction and it was observed that different benzyl alcohols were converted to desire amides with good to excellent efficiency. This reaction was performed in the presence of amine hydrochloride salts, tert-butyl hydroperoxide (TBHP), and Ca_2_CO_3_ in acetonitrile (CH_3_CN) under nitrogen atmosphere. CN-Pr-β-CD/Cu(II) can be recycled and reused five times without significant reduction in reaction efficiency.

## Introduction

Amides are one of the most important functional groups in various industrial and scientific research fields such as polymers, drugs, chemical raw materials, biomolecules, and natural products^1–4^. Numerous methods have been reported for the synthesis of these valuable materials^5,6^. Traditionally, the vast majority of amides were prepared through interaction of amines with active or inactive carboxylic acid derivations^7,8^. In addition, reactions such as Schmidt^9^, Ugi^10^, Staudinger^11^, and Ritter^12^, were used to synthesize amides. Risks or negative effects of these methodologies have led to the creation of lower risk protocols. Recently, various modified procedures like oxidative amidations reactions^13^, primary amides transamination via amines^14^, alkynes hydroamination^15^, and amines acylation have been described^16^. Among established synthetic methods, one of the greenest procedures is amide formation via tandem oxidative amidation of benzylic alcohols by transition metal catalysts. Various metals such as “Ru”^17^, “Rh”^18^, “Zn”^19^, “Fe”^20^, “Au”^21^, “Ir”^22^, and “Cu”^23^ can be used in this reaction as catalysts. Among the reported metals, copper due to the natural abundance, high activity, and low price was considered as an attractive option to catalyze these reactions^24^. However, one of the main problems of these metals as catalysts is the difficulty of their separation from the reaction medium. To overcome this problem, these metals are usually coordinated on a solid surface via appropriate ligands. In this regard, various materials like graphene oxide, Nitrogen-doped (N-doped) graphene, hexagonal boron nitride, metal chalcogenide, and graphitic carbon nitride (g-C_3_N_4_) can be used^25^. g-C_3_N_4_ as a metal-free polymeric structure with two-dimensional layered morphology, has attracted special attention due to the features such as chemical and thermal stability, wide surface area, and biocompatibility^26^. CN, as one of the most important forms of g-C_3_N_4_ has many applications in fields of dyes photodegradation^27^, solar energy^28^, supercapacitors^29^, water splitting^30^, sensors^31^, hydrogen evolution^32^, and catalysts^33^. Ligands also play a very important role in the coordination of metal on a solid support, and in this regard, β-Cyclodextrin (β-CD) has been considered. β-CD is cyclic oligosaccharides, made up from seven D-glucopyranose parts. β-CD conical cylinder framework has an inner lipophilic space as well as an outer hydrophilic one^34^. During recent years, β-CD special features and ability of creation complex have attracted major attention in the areas of food^35^, medicine^36^, textile^37^, cosmetology^38^, enantiomeric separation^39^, environmental remediation^40^, chemical sensing^41^, and catalysis^42^. Combining CN with various metal supported β-CD structures for catalytic and photocatalytic reactions have become an attractive strategy in improving the performance of reactions^43,44^. For example, in the study performed by Zuo and et al. in 2020, Au nanoparticles were coordinated on β-CD modified g-C_3_N_4_. This nanocomposite was used in the photocatalytic production of hydrogen peroxide^43^. Herein, CN was functionalized by β-CD/Cu(II) and this modification was performed by 1,3-dibromopropane as a linker. CN-Pr-β-CD/Cu(II) was used as a catalyst in tandem oxidative amidation of benzylic alcohols and various derivatives were synthesized with good to excellent efficiencies.

## Experimental

### Reagents and instruments

In this study, all used materials were purchased from reputable international companies, including Flucka (Switzerland) and Merck (United States). The reaction conditions were controlled by Thin Layer Chromatography (TLC) by utilizing a 0.2 mm F254 Merck silica gel coated on aluminum plates (United States). Also, the accuracy of forming the products was investigated using the melting point with the electrothermal 9100 device (United Kingdom). 800 IR 100 device from Shimadzu company was used for the record Fourier transform (FT)-IR spectra to identify catalysts and reaction products (Japan). This analysis was performed in the range of 400–4000 cm^−1^. The X-ray diffraction (XRD) analysis was used to identify the crystal structure of the catalyst components and PANalytical X-PERT-PRO MPD apparatus was used for this purpose (Netherlands). This analysis was evaluated in the range of 2θ, 0.5° to 10° and 10° to 80°. Energy Dispersive X-ray Spectroscopy (EDS) was performed by ZEISS-Sigma VP (Germany) in order to examine the elements of the catalyst. Field emission scanning electron microscopy (FE-SEM) by MRIA3 TESCAN-XMU instrument (Czechia), Thermogravimetric analysis (TGA) by STA504 device (United States) and inductively coupled plasma optical emission spectroscopy (ICP-OES) by VISTA-PRO (United States) were used to evaluate the morphology, thermal stability and recyclability of the catalyst, respectively. N_2_ adsorption/desorption isotherms was used to investigate the surface area of the synthesized catalyst. For this purpose, 2020 ASAP™ apparatus made by American company micromeritics was used. Transmission electron microscopy (TEM) imaging was performed using the EM10C-100 kV device made by the German company ZEISS to investigate the dispersion of metal particles in the catalyst structure.

### Preparation of Bulk g-C_3_N_4_

First 5 g of white melamine powder heated for 4 h at 550 °C by laboratory furnace under static air. This process was done with a temperature ramp of 2.5 °C min^−1^. Finally, the resulting yellow solid was grinded to powder particles^45^.

### Preparation of CN

The procedure utilized to prepare the nanosheet form of the g-C_3_N_4_ was introduced by Li and et al.^46^. First, 1 g of the synthesized g-C_3_N_4_ was dispersed in a mixture containing 20 ml of nitric acid (65 wt%) and 20 ml of sulfuric acid (98 wt%) for 2 h at ambient temperature. Then, 1 L of deionized water was added to dilute the mixture. The precipitate was collected and washed several times with deionized H_2_O, and finally dried in an oven at 60 °C. In the next step, 1 mg of g-C_3_N_4_ was dispersed in 100 ml of H_2_O/isopropanol (1:1) for 6 h by an ultrasonic bath. Eventually, a centrifuge with speed of 5000 RPM was used to separate the synthesized CN.

### Preparation of CN-Pr-Br

1 g of the synthesized CN was added to 25 ml of dry toluene and the mixture was sonicated for 1 h. Then, 1 mmol of sodium iodide and 2.02 ml of 1,3-dibromopropane were added to the dispersed mixture and the reaction suspension was refluxed under nitrogen atmosphere for 2 days. The functionalized CN was separated by centrifugation and dried at ambient temperature.

### Preparation of β-CD/Cu(II)

The copper and β-CD complex was constructed using a method reported by Kaboudin and et al.^47^. Initially, 1 mmol of β-CD was added to 250 ml beaker including 50 ml of 1 M sodium hydroxide solution, and the mixture was stirred until a clear solution was formed. Afterward, 75 ml solution contain 0.04 M of CuSO_4_(H_2_O)_5_ salt was added to the clear solution prepared in the previous step. The dark blue solution obtained at this stage was stirred at 25 °C for 6 h. After this time, the mixture was passed through filter paper to separate the excess copper as copper (II) hydroxide. Finally, 400 ml of ethanol was added to the remaining solution to form a pale blue suspension. The precipitate was separated by the Buchner funnel and after washing with ethanol, dried at 60 °C.

### Preparation of CN-Pr-β-CD/Cu(II)

0.1 g of the functionalized CN and 20 ml of dry toluene were added to a 50 ml flask and sonicated for 30 min. Then, 0.1 g of β-CD/Cu(II) complex, 0.1 mmol of sodium iodide and 0.1 mmol of potassium carbonate were added to the reaction vessel and refluxed under N_2_ for 2 days. Eventually, the precipitate was collected by centrifugation, washed several times with water and ethanol, and dried in a 60 °C oven.

### General Procedure for the Tandem oxidative amidation of benzylic alcohols catalyzed by CN-Pr-β-CD/Cu(II)

To a 10 ml round bottom flask, 1.5 mmol of benzyl alcohol, 1 mmol of hydrochloride salt of amine, 1/1 equivalents of calcium carbonate, 3 equivalents of TBHP, 15 mg of CN-Pr-β-CD/Cu(II), and 3 ml of CH_3_CN were added. The mentioned materials were then refluxed under N_2_ for 3 h at 80 °C. After the completion of the reaction was confirmed using TLC, the catalyst was separated by filter paper and washed through ethanol for subsequent use. Eventually, the CH_3_CN was evaporated by a rotary, and the product was extracted (H_2_O/ethyl acetate) and recrystallized (by ethanol) for further purification.

## Results and discussion

### Catalyst characterization

**Figure 1:**
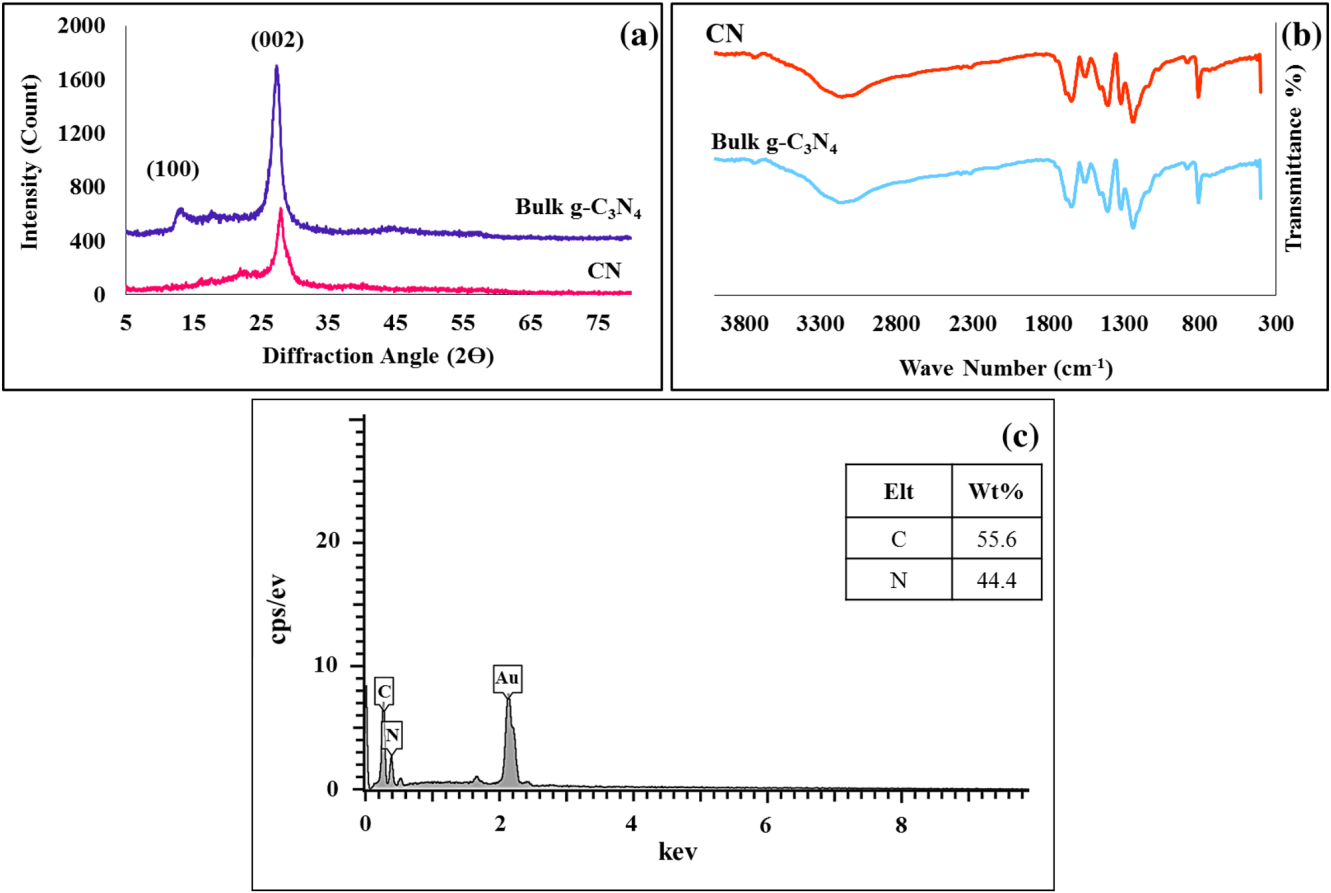
(**a**) XRD patterns and (**b**) FT-IR spectra of bulk g-C_3_N_4_ and CN. (**c**) EDS spectrum of the CN.

In this study, CN-Pr-β-CD/Cu(II) was prepared by a facile procedure. Initially, CN with two-dimensional morphology were prepared via exfoliation in liquid phase and then the layers were separated by ultrasonic treatment. XRD, FT-IR and EDS analysis were used to confirm the correct formation of CN and they were demonstrated in Fig. 1. According to information obtained from XRD analysis, bulk g-C_3_N_4_ has been converted to nanosheets form of the g-C_3_N_4_. The XRD spectrum of bulk g-C_3_N_4_ has two main peaks in 2θ = 27.4° and 13.1° which is related to the interaction between conjugated aromatic part of the system and tri-s-triazine units of the structure (Fig. 1a). After exfoliation, the peak intensity of the (002) decreases and its 2θ position shifts from 27.4° to 27.8°, which may be related to the distance between the g-C_3_N_4_ layers. Also during this process, another g-C_3_N_4_ peak in 2θ = 13.1° was removed^26^. In addition, the value of d-spacings for the peaks observed in 27.4° and 13.1° for bulk g-C_3_N_4_ is 0.325 nm and 0.675 nm, respectively^48^. After the exfoliation process and CN synthesis, the value of d-spacings for the peak in 2θ = 27.8° is equal to 0.321 nm. The slight decrease observed in d-spacings during peak displacement from 27.4° to 27.8° is due to the flattening of the undulated layers in g-C_3_N_4_. In other words, the heat and oxidation applied to convert bulk g-C_3_N_4_ to nanosheets, compress the CN monolayers together and shorten the interplane distance of the CN sample^49^.

**Figure 2:**
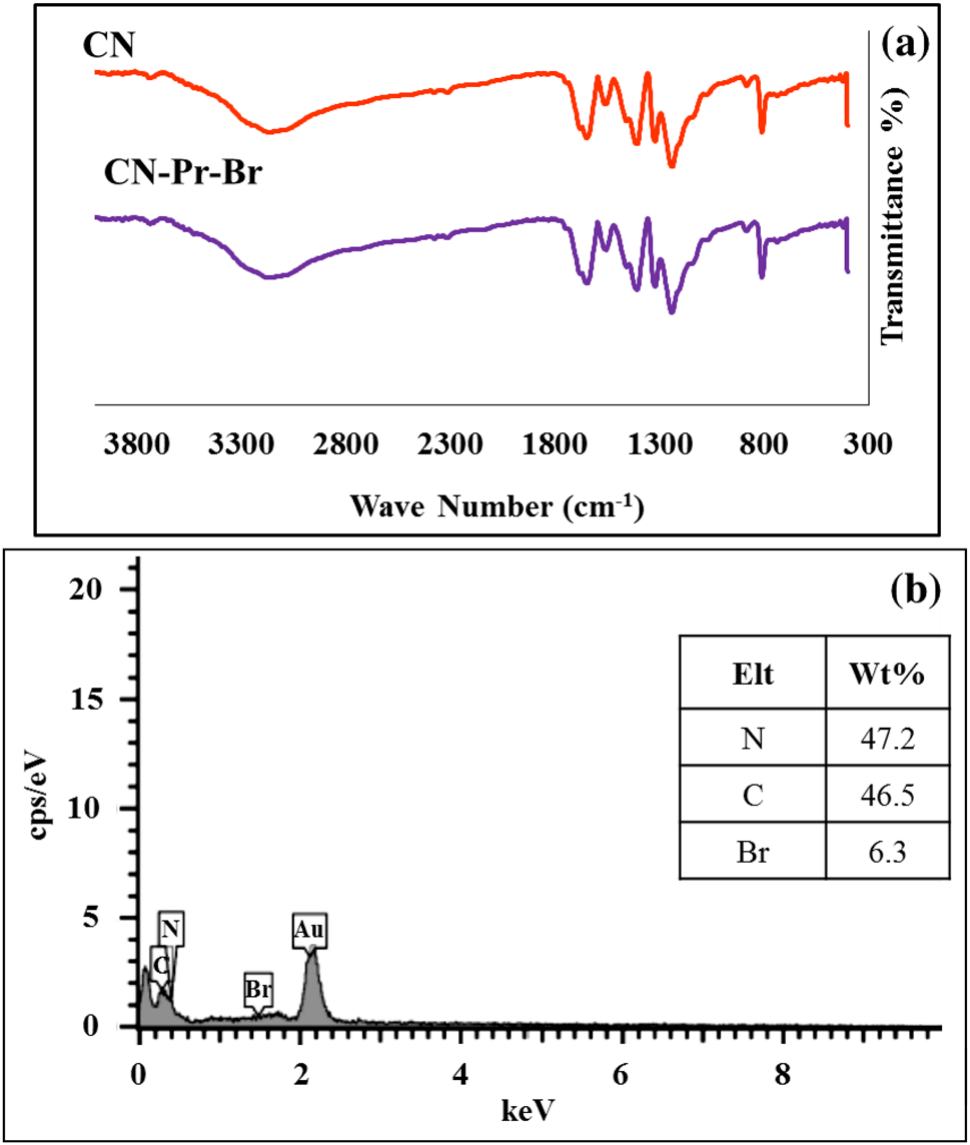
(**a**) FT-IR spectra of CN and CN-Pr-Br. (**b**) EDS analysis of CN-Pr-Br.

**Figure 3:**
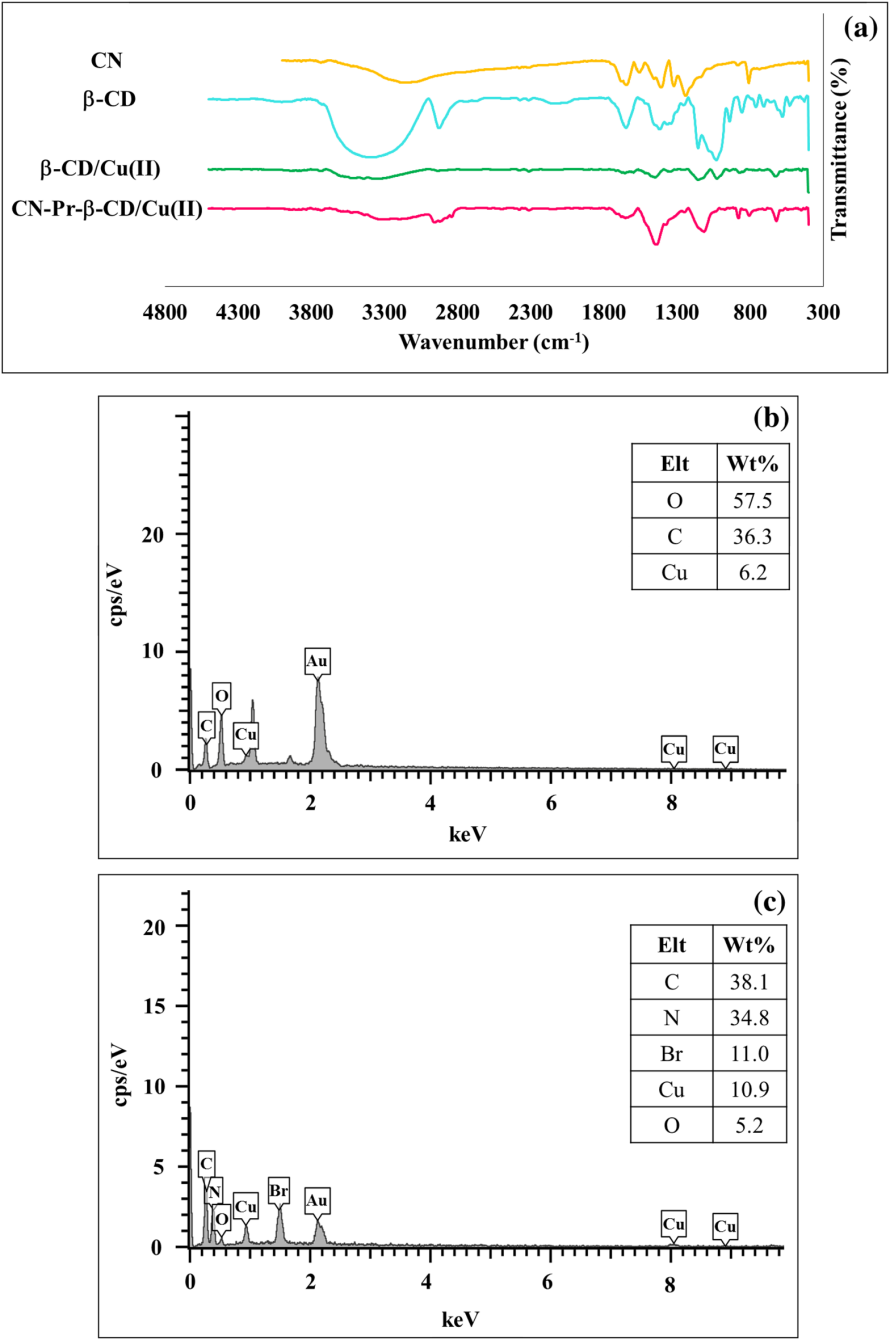
(**a**) FT-IR spectra of CN, β-CD, β-CD/Cu(II), CN-Pr-β-CD/Cu(II), (**b**) EDS analysis of β-CD/Cu(II), and (**c**) EDS analysis of CN-Pr-β-CD/Cu(II).

The FT-IR spectra of bulk g-C_3_N_4_ and CN are similar to each other. According to Fig. 1b, the peak observed in the range of 3000−3500 cm^-1^ was related to N–H present on the g-C_3_N_4_. Also, the observed peaks in the range of 1614 cm^−1^ and 1550 cm^−1^ corresponded to the stretching vibration of C=N. C–N stretching peaks were located in 1406 cm^−1^, 1319 cm^−1^, and 1234 cm^−1^. Also sharp peak at 808 cm^−1^ was related to the breathing vibration of the tri-s-triazine components. EDS confirmed the presence of carbon and nitrogen in the g-C_3_N_4_ structure (Fig. 1c). Subsequently, 1,3-dibromopropane was used to modify the CN and the linker binding process was examined by FT-IR and EDS analyzes. As can be seen in Fig. 2a, the FT-IR spectrums of CN-Pr-Br and linker-free CN were identical. On the other hand and based on Fig. 2b, EDS analysis confirmed the presence of the Br atoms. Finally, to increase the stability of β-CD/Cu(II) and also to promote the catalytic properties and dispersion of CN in water, CN-Pr-β-CD/Cu(II) were synthesised. As presented in Fig. 3a, the main peaks for the β-CD structure, including the sharp peak at 2920 cm^−1^ and the wide peak at 3369 cm^−1^, can be related to the stretching vibrations of the C-H and OH bonds, respectively. In addition, glucose peaks, including C–C, C-O, and C–O–C, are found in the areas of 1027 cm^−1^, 1155 cm^−1^, 1340 cm^−1^, 1417 cm^−1^, 1647 cm^−1^. FT-IR spectrum of copper (II) supported β-CD, confirm complex formation of the β-CD with copper (II). According to this spectrum, the absorption frequencies in the β-CD structure were shifted to lower frequencies. This shifts to lower frequencies were confirmed the β-CD complexation with copper. Eventually, by comparing the CN-Pr-β-CD/Cu(II) spectrum with the spectra of raw materials in previous stages, it can be concluded that the presence of absorption bands in 2948 cm^−1^ are related to the CH stretching vibrations in the β-CD molecule and the 1,3-dibromopropane linker. Moreover, the absorption band at 1110 cm^−1^ was confirmed the formation of a CO bond in the final catalyst. The main adsorption bands relevant to the structure of CN and β-CD are exist in final structure of the catalyst.

**Figure 4:**
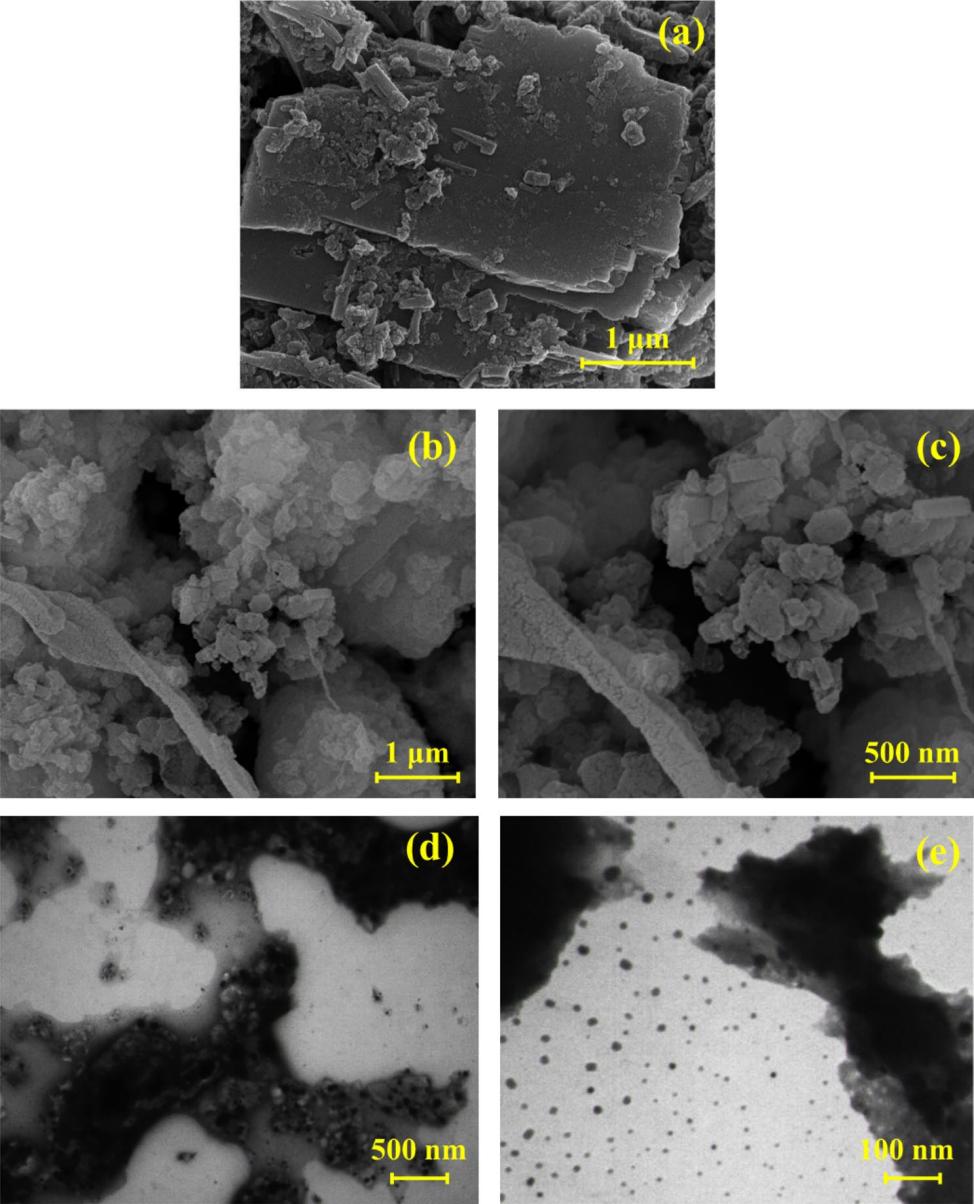
FE-SEM images of (**a**) CN, (**b**,**c**) CN-Pr-β-CD/Cu(II) and, (**d**,**e**) TEM images of CN-Pr-β-CD/Cu(II).

In the next step, the elements in the structure of the β-CD/Cu(II) and CN-Pr-β-CD/Cu(II) were examined by EDS analysis. As can be seen in Fig. 3b,c, β-CD/Cu(II) contains carbon, oxygen, copper and on the other hand, CN-Pr-β-CD/Cu(II) includes carbon, nitrogen, bromine, oxygen and copper. ICP-OES was also utilized to determine the amount of copper in the structure of the CN-Pr-β-CD/Cu(II) and the presence of 12.7% copper in the catalyst structure was confirmed. FE-SEM and TEM imaging were performed to evaluate the structure and morphology of the catalyst. The FE-SEM images of CN and CN-Pr-β-CD/Cu(II) are shown in Fig. 4a and Fig. 4b,c respectively. Accordingly, CN plates and the functionalization process were well observed. On the other hand, as seen in the TEM image of CN-Pr-β-CD/Cu(II) in Fig. 4d,e, acceptable dispersion for metal particles were observed in the catalyst structure.

**Figure 5:**
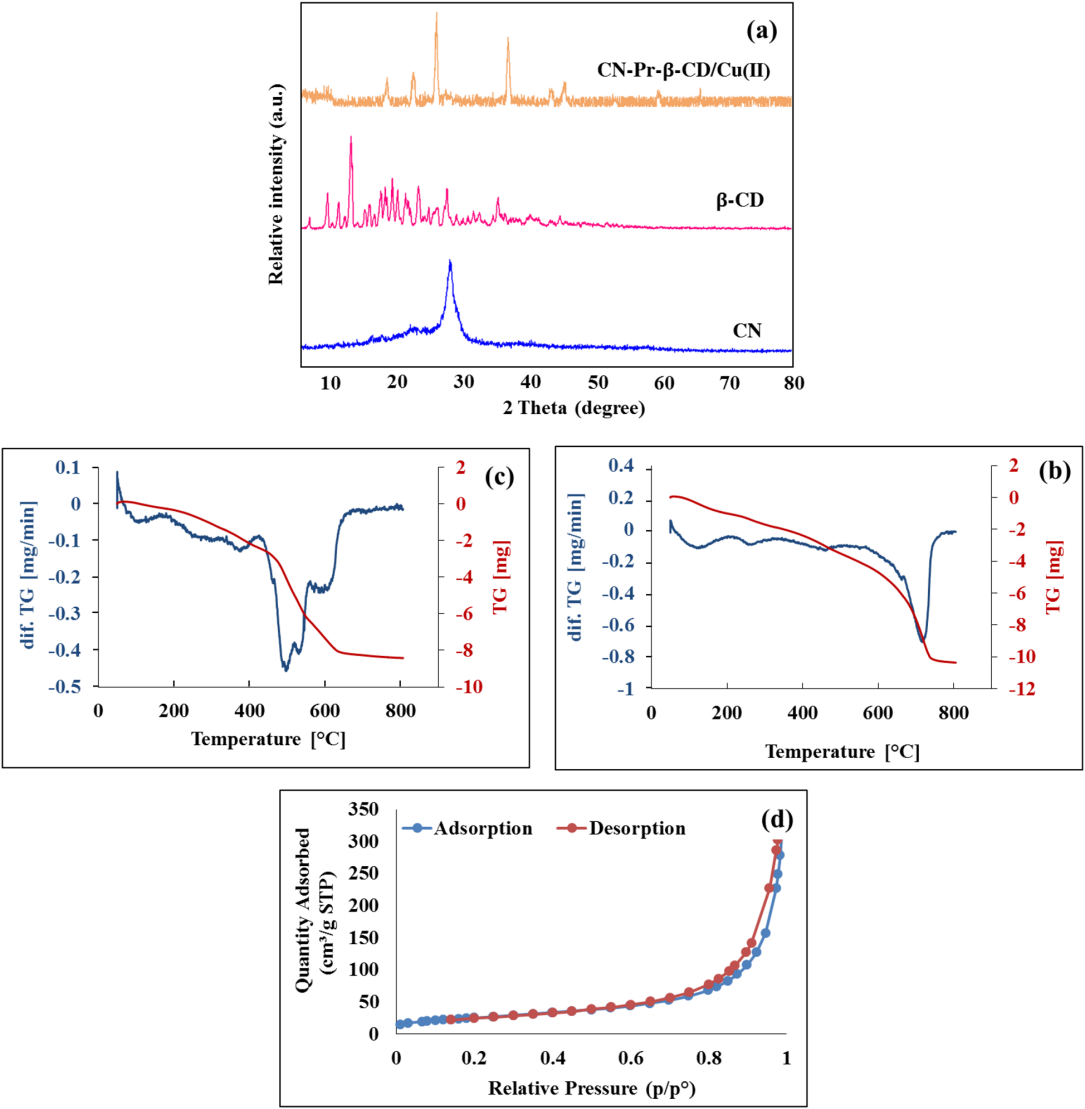
(**a**) XRD patterns of CN, β-CD, CN-Pr-β-CD/Cu(II), (**b**) TGA patterns of CN, (**c**) TGA patterns of CN-Pr-β-CD/Cu(II) and, (**d**) N_2_ adsorption–desorption isotherms of CN-Pr-β-CD/Cu(II).

**Figure 6:**
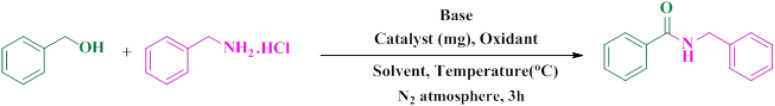
Model reaction to optimize the tandem oxidative amidation of benzylic alcohols by CN-Pr-β-CD/Cu(II) catalysts.

The XRD patterns of CN, β-CD, and CN-Pr-β-CD/Cu(II) were indicated in Fig. 5a. As shown in spectrum of the CN-Pr-β-CD/Cu(II), diffractions peaks related to the structure of β-CD were observed in the XRD diagram of the catalyst which confirm the functionalization of the CN. Furthermore, the diffractions peak at 2θ about 60.0° is related to the copper in the structure of the CN-Pr-β-CD/Cu(II) catalyst. TGA used to investigate the thermal stability of the synthesized samples. The TGA diagram for CN and the final catalyst was shown in Fig. 5b,c respectively. According to the Fig. 5b, the main mass loss of the CN begin at 600 °C. Figure 5c shows the CN-Pr-β-CD/Cu(II) TGA curve. In this diagram, three main mass reduction steps were observed, 3% mass reduction observed in the range of 201–106 °C, which can be related to the evaporation of water and the exit of solvents trapped in the structure. The 16% mass drop in the range of 401–203 °C was related to the breakdown of the linker and part of the β-CD. Finally, the mass drop at temperatures above 400 °C was due to the complete loss of β-CD and CN. Finally, it can be concluded that the functionalization process occurred well and during this process, the thermal stability of β-CD/Cu(II) increased. BET analysis was also performed to evaluate the surface area of the synthesized catalyst via nitrogen adsorption–desorption equilibrium (Fig. 5d). According to the obtained results, the BET surface area of the synthesized catalyst was 90.2127 m^2^/g. The catalytic capacitance of CN-Pr-β-CD/Cu(II) was reconnoitered for the preparation of amide derivatives via oxidation of benzylic alcohols with the amine hydrochloride salts. An overview of this process can be found in Fig. 6. To perform the reaction, the effective parameters in the reaction were first optimized to achieve the highest efficiency. As shown in Table 1, the effect of different factors on the reaction including catalyst, oxidizing agent, base, temperature and solvent was investigated.

**Table 1:** Optimization of the reaction condition for the one-pot direct oxidative amidation.

Entry	Solvent	Oxidant	Amount of oxidant (equivalent)	Base	Amount of catalyst (mg)	Temperature (°C)	Yield (%)^a^
1	CH_3_CN	–	–	–	20	80	–
2	CH_3_CN	TBHP	4	–	20	80	–
3	CH_3_CN	–	–	CaCO_3_	20	80	–
4	CH_3_CN	TBHP	4	CaCO_3_	20	80	80
5	CH_3_CN	TBHP	4	CaCO_3_	–	80	–
6	CH_3_CN	TBHP	3	CaCO_3_	20	80	90
7	CH_3_CN	TBHP	3	CaCO_3_	25	80	83
8	CH_3_CN	TBHP	3	CaCO_3_	15	80	95
9	CH_3_CN	TBHP	3	CaCO_3_	10	80	80
10	CH_3_CN	H_2_O_2_	3	CaCO_3_	15	80	30
11	CH_3_CN	O_2_	–	CaCO_3_	15	80	25
12	CH_3_CN	TBHP	3	CaCO_3_	15	100	86
13	CH_3_CN	TBHP	3	CaCO_3_	15	60	80
14	CH_3_CN	TBHP	3	Na_2_CO_3_	15	80	Trace
15	CH_3_CN	TBHP	3	K_2_CO_3_	15	80	–
16	Toluene	TBHP	3	CaCO_3_	15	80	20
17	DMF	TBHP	3	CaCO_3_	15	80	40
18	DMSO	TBHP	3	CaCO_3_	15	80	55

Reaction condition: benzylamine hydrochloride (1.0 mmol), benzyl alcohol (1.5 mmol), catalyst (CN-Pr-β-CD/Cu(II)), solvent (3 mL), Base (1.1 equiv), oxidant (70 wt % in H_2_O, 3 equiv), under N_2_ atmosphere.

^a^Isolated yield.

The model reaction performed by 20 mg CN-Pr-β-CD/Cu(II) catalyst and 3 equivalents of TBHP in the presence of calcium carbonate. Based on the observed results, the presence of both base and oxidizing agents is necessary for the reaction to take place (Rows 1–3, Table 1). This reaction was accomplished at 80 °C in CH_3_CN solvent under N_2_ atmosphere (Row 4, Table 1). Under these conditions, *N*-benzylbenzamide was obtained with 80% efficiency. Then, the effect of various factors including the amount of oxidizing agent, the type of oxidizing agent, the quantity of catalyst, the reaction temperature, the type of base, and the reaction solvent were investigated. Reducing the amount of TBHP to 3 equivalents, increases the production efficiency of the target product (90%) (Row 6, Table 1). In the next step, the amount of catalyst used in the reaction was investigated. As shown in Row 8 of Table 1, 15 mg of CN-Pr-β-CD/Cu(II) catalyst had the best effect at reaction efficiency (95%). Hydrogen peroxide (H_2_O_2_) and oxygen (O_2_) were used as green oxidizing agents in this reaction (rows 10–11, Table 1). The best yield of the desired amide product was obtained by TBHP under nitrogen atmosphere. In the next step, different temperatures in the range of 60 to 100 were examined (rows 8, 12 and 13, Table 1) and it was observed that increasing the reaction temperature to 100° C reduces the reaction efficiency to 86% (Row 12, Table 1). Decreased in the reaction efficiencies may be related to the oxidation of benzyl alcohol to the related benzoic acid as a by-product that occurred at higher temperatures (benzoic acid by-product formation was confirmed by TLC). Further studies in this area have shown that the best temperature for the reaction was 80 °C. Various bases including Na_2_CO_3_, K_2_CO_3_ and CaCO_3_ were used to optimize the reaction conditions (rows 8, 14 and 15, Table 1). Among them, calcium carbonate minimized the adverse oxidation reaction of amine via slow deprotonation of the amine hydrochloride salt. As a result, the yield of the target product was increased. Eventually, the model reaction was done in CH_3_CN, toluene, dimethylformamide (DMF) and dimethyl sulfoxide (DMSO) solvents (rows 8, 16, 17 and 18, Table 1) and the best yield for *N*-benzylbenzamide was obtained in CH_3_CN solvent.

To demonstrate the potential and importance of using the CN-Pr-β-CD/Cu(II) catalyst in the tandem oxidative amidation of benzylic alcohols, the model reaction was performed under optimal condition with the presence of other catalysts such as copper sulfate salt, CN, β-CD, CN/Cu(II) and copper (II) supported β-CD (Table 2). Based on the obtained results in the mentioned table, the CN-Pr-β-CD/Cu(II) catalyst had the highest efficiency in the amide synthesis reaction. The presence of copper is an essential factor in promoting this reaction.

**Table 2:** Comparison of the catalytic performance of CN-Pr-β-CD/Cu(II) with other catalysts in synthesis of amide through the oxidative amidation of benzylic alcohols.

Entry	Catalyst	Yield (%)^a^
1	CN	–
2	CuSO_4_.5H_2_O	30
3	β-CD	–
4	β-CD/Cu(II)	45
5	CN/Cu(II)	75
6	CN-Pr-β-CD/Cu(II)	95

Reaction condition: benzylamine hydrochloride (1.0 mmol), benzyl alcohol (1.5 mmol), Catalyst (15 mg), CH_3_CN (3 mL), CaCO_3_ (1.1 equiv), TBHP (70 wt % in H_2_O, 3 equiv).

^a^Isolated yield.

After determining the optimal conditions, various amides were synthesized by different benzylic alcohols and amine salts and the results are demonstrated in Table 3. Based on the information shown in Table 3, the oxidative amidation of benzylic alcohols with different aliphatic and aromatic amine hydrochloride salts (types 1, 2, and 3) and different type of benzylic alcohols including electron donor and withdrawing groups has been studied. In all cases the desired amide was obtained with the appropriate yield.

**Table 3:** Direct oxidative amidation of benzyl alcohols with amine salts.


Entry	Alcohol	Amine salt	Product	Yield (%)^a^	Mp. (^o^C) (Ref.)
1		a		95	108–110^50^
2		a		90	162–164^51^
3		a		95	101–103^52^
4		a		93	132–134^53^
5		a		75	139–142^54^
6		a		65	95–97^55^
7		b		90	160–162^56^
8		b		85	200–202^57^
9		b		90	158–159^58^
10		c		75	73–75^59^
11		d		85	130–133^60^
12		d		80	100–103^61^
13		e		87	128–130^62^
14		e		85	173–175^63^
15		e		90	165–166^63^
16		f		93	72–74^63^
17		f		87	104–106^64^
18		f		94	67–69^65^
19		g		75	Yellow oil^63^

Reaction condition: amine hydrochloride (1.0 mmol), benzyl alcohols (1.5 mmol), Catalyst (CN-Pr-β-CD/Cu(II)) (15 mg), CH_3_CN (3 mL), CaCO_3_ (1.1 equiv), TBHP (70 wt% in H_2_O, 3 equiv).

^a^Isolated yield.

**Figure 7:**
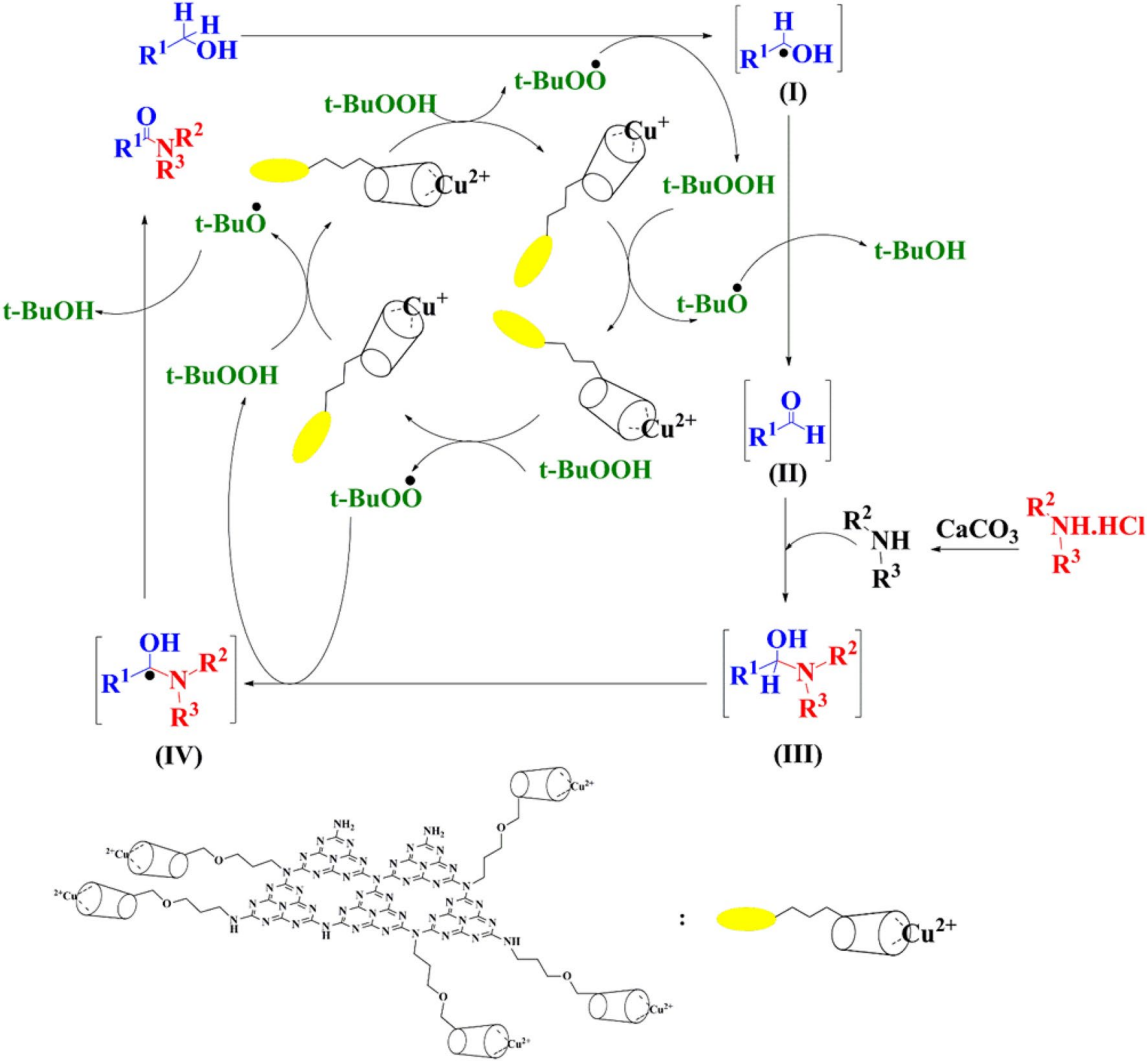
The proposed mechanism for synthesis of amide through the oxidative amidation of benzyl alcohols by CN-Pr-β-CD/Cu(II) catalyst.

The outline of the possible mechanism for tandem oxidative amidation of benzylic alcohols was indicated in Fig. 7. TBHP is an excellent source of oxygen and can be used for oxidation reactions after activation by a suitable transition metal complex^66^. In this regard, benzyl alcohol in the presence of CN-Pr-β-CD/Cu(II) and TBHP was oxidized to aldehyde through a radical mechanism and tert-butanol was released. In addition, tert-butylperoxyl and tert-butoxyl radicals were also produced through Eqs. (1) and(2), respectively^20,67^. In the next step, calcium carbonate as the base separates the proton from the amine hydrochloride salt, and the resulting free amine reacts with the aldehyde and eventually producing the hemiaminal intermediate (III)^20,68^. Reduction of copper (II) ions in the CN-Pr-β-CD/Cu(II) catalyst leads to the production of tert-butyl peroxy radical. Subsequently, the hemiaminal was converted to intermediate (IV) in the presence of the activated tert-butyl peroxy radical. Finally, intermediate (IV) by the CN-Pr-β-CD/Cu(II)-TBHP catalytic system produces the desired amide through a radical mechanism by removing the tert-butanol. Then, to ensure the radical process of the reaction mechanism, 2,6-Di-tert-butyl-4-methylphenol (BHT) was used as a radical scavenger in the model reaction and no product was observed during the reaction. From this, it can be concluded that the oxidation reaction of benzyl alcohols has proceeded in a radical way.

1 $${\text{Cu}}^{2+} + t-{\text{BuOOH}} \to {\text{Cu}}^{+} + t-{\text{BuOO}}^{\cdot} + {{\text{H}}^{+}}$$

2 $${\text{Cu}}^{+} + t-{\text{BuOOH}} + {{\text{H}}^+ } \to {\text{Cu}}^{2+} + t-{\text{BuO}}^{\cdot} + {{{\text{H}}_{2}}{\text{O}}}$$

**Figure 8:**
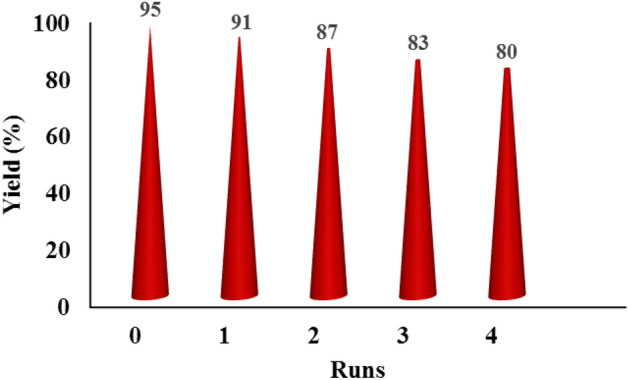
Reusability of the CN-Pr-β-CD/Cu(II) catalyst in the synthesis of amide through the oxidative amidation of benzyl alcohols.

The ability to recycle and reuse the catalyst is one of the most important aspects of catalyst design. In this regard, the CN-Pr-β-CD/Cu(II) catalyst was separated from the other components of the reaction by filter paper and dried after washing with ethanol and used again in model reaction. According to the obtained result in Fig. 8, the prepared catalyst can be reused up to 5 times in the model reaction. Catalyst leaching was also evaluated and according to the obtained results from ICP-OES, Cu% decreased from 12.7% to 12.65% after five cycles. Following the study of catalytic properties, a comparison between the synthesized catalyst and previous articles was made. Based on information summarized in the Table 4, the CN-Pr-β-CD/Cu(II) catalyst is competitive with other reports in product efficiency, reaction time and conditions, as well as the ability to recyclability the catalyst.

**Table 4:** Comparison between the catalytic efficiency of the CN-Pr-β-CD/Cu(II) with the reported catalysts for the amide syenthesis through the oxidative amidation of benzyl alcohols.

Entry	Catalyst	Oxidizing agent	Base	Reaction time (h)	Yield (%)	References
1	Au/DNA	O_2_	LiOH.2H_2_O	12	80	^69^
2	Fe_3_O_4_@Fe(OH)_3_	TBHP	CaCO_3_	6	89	^20^
3	CuO	TBHP	CaCO_3_	4	70	^70^
4	MagCNTs@SiO_2_-linker-CuI	T-Hydro	Na_2_CO_3_	6	83	^71^
5	Au6Pd/resin	O_2_	NaOH	12	83	^72^
6	Diacetoxyiodobenzene (DIB)	TBHP	–	10	69	^73^
7	Fe(NO_3_)_3_	O_2_/TBHP	CaCO_3_	16	78	^74^
8	(PyPS)_3_PW_12_O_40_	TBHP	–	12	80	^75^
9	CN-Pr-β-CD/Cu(II)	TBHP	CaCO_3_	3	95	This work

## Conclusions

In this study, CN-Pr-β-CD/Cu(II) was synthesized and evaluated as an effective catalyst for the tandem oxidative amidation of benzylic alcohols. Catalyst synthetic processes were performed via modification of CN by 1,3-dibromopropane and β-CD/Cu (II) respectively. CN-Pr-β-CD/Cu(II) was evaluated and identified by using analysis such as FT-IR, XRD, FE-SEM, EDS, TGA, ICP-OES, BET, and TEM. The mentioned catalytic reaction was performed in the presence of amine hydrochloride salts, TBHP, and Ca_2_CO_3_ in CH_3_CN solvent. The transition metal by activating the TBHP plays a key role in the progress of the reaction. According to the obtained results, the tandem oxidative amidation of benzyl alcohols with various aromatic and aliphatic amine hydrochloride salts (types I, II and III) as well as various benzyl alcohols including electron withdrawing and donor groups have been investigated. In all cases the desired amides were obtained with the appropriate yields.
